# Well-child care delivery in the community in China: Related factors and quality analysis of services

**DOI:** 10.1371/journal.pone.0190396

**Published:** 2018-01-23

**Authors:** Pan Mao, Hui Feng, Shuang Xu, Jianghong Liu, Huayan Li, Yaying Zhang, Yiyan Ye

**Affiliations:** 1 Henan Provincial People’s Hospital, Zhengzhou, Henan, China; 2 Xiangya School of Nursing, Central South University, Hunan, China; 3 Health Service Research Center, Central South University, Hunan, China; 4 School of Nursing and School of Medicine, University of Pennsylvania, Philadelphia, United States of America; 5 Peking University People’s Hospital, Peking, China; 6 The Second Xiangya Hospital of Central South University, Changsha, Hunan, China; 7 Bei Nuo Hospital, Changsha, Hunan, China; Brown University, UNITED STATES

## Abstract

Well-child health care services are essential for maintaining optimum child health and development. This study’s aim was to evaluate the quality of such services and identify factors affecting service quality from the perspective of well-child health care providers located in China’s Hunan Province. To achieve this, a qualitative descriptive method was employed, with 22 well-child health care providers being recruited, using purposive sampling, from among the provinces’ government community health centers. The participants completed individual semi-structured interviews lasting approximately 25–30 minutes that were designed to obtain their views on well-child health care administration in the province. Then, the interview transcripts were analyzed thematically. The main finding was that participants felt that the delivery of well-child health care services in Hunan Province is insufficient. Factors they mentioned as negatively affecting the delivery of such services included the region’s fragmented primary health care system, inadequate attention to this issue from parents and community health care center managers, and a lack of specialized well-child care knowledge. Thus, currently, well-child health care is not being successfully implemented in Hunan Province; consequently, in order to successfully implement well-child health care in this region, community health care centers should invest more resources and funding, particularly into education programs for well-child health care providers.

## Introduction

In 2013, the number of children in China aged 0–14 years reached 220 million, meaning this demographic accounted for 16.4% of the country’s population [1]. As a result of this large number of children, child health has become a major problem affecting the national economy’s development [2]. The proportion of infants in the country receiving breast milk from their mothers is declining, the proportion of children left at home alone is increasing, and most are being cared for by their grandparents. However, most grandparents do not know how to take care of children in an appropriate and scientific manner. They just feed children, not educate children. Consequently, some Chinese children have developmental problems (with some even suffering from psychological problems) and the nutritional status of rural- and urban-dwelling children continues to differ considerably [3,4]. Well-child health care is an important aspect of the 12 national basic public health care services. Specifically, these 12 public health care services are: residents’ health records management; health education; immunization; well-child care for children aged 0–6 years, including mental health management; elderly health management; high blood pressure management; diabetes management; severe mental illness management; infectious disease and public health management; health supervision and management; Chinese medicine management; and tuberculosis patient management. Well-child care services incorporate regular monitoring and evaluation of children’s health status, providing guidance and implementing interventions that can maintain children’s quality of health, and assessing consequential factors such as children’s growth and development. In particular, well-child health care promotes children’s health and development through the application of a comprehensive health tracking system [5], and also monitors child growth, addresses behavioral problems, assesses children’s likelihood of fulfilling their developmental potential, conducts early development and management, and performs disease diagnosis and treatment [6]. However, the increasing demand for child health care in China is challenging the capacity of well-child health care providers (WHCPs). WHCPs are the medical-related professionals who typically have the most frequent contact with children and families; therefore, they play an important role in the delivery of these services [7]. However, very little research has evaluated the quality of well-child health care services from the perspective of WHCPs. Consequently, conducting an investigation from this perspective may facilitate the evaluation of well-child health care delivery in China and could also identify factors that affect service quality and availability.

If we contrast the situation in China with that in other countries, we find some interesting variations. In the US, general pediatricians typically conduct well-child health care work for people aged < 21 years. During routine well-child visits, pediatricians emphasize the identification and addressing of family psycho-social, developmental, and behavioral issues [8–11]; specifically, US pediatricians’ well-child health care capacity has been described as comprising the following: (1) performing health supervision; (2) performing developmental surveillance of acquired milestones and school performance; (3) assessing child and family psycho-social status; (4) performing care coordination; and (5) offering physical examination, immunization, and screening services [6, 12,13]. Meanwhile, in Australia, primary- and secondary-prevention health services for children and families are provided by maternal and child health (MCH) nurses; these are registered nurses who have specialist qualifications in child and family health [14]. Before beginning work as an MCH nurse, most of these nurses obtain a bachelor’s or master’s degree and undergo specialized training to obtain a community nurse certificate. This additional education has an important, positive effect on the successful delivery of child health prevention services [15].

Returning to China, a well-child care service has been available in the country for eight years (since 2009). In an attempt to promote the provision of this well-child care service, in 2011 the *Country Public Health Service Specification* (for 0–6-year-old children) and the *Ministry of Well-child Health Care Guidelines* were published. These guidelines state that children should be screened regularly and that every screening should be logged in each child’s health records [16,17]. As part of this system, primary community health centers are tasked with assessing growth and development, hearing and vision, and psychological, language, and social skills [16]; therefore, these centers play a major role in providing care to children. Further, the *National Maternal and Child Health Care* guidelines stress that WHCPs should work with nurses, general practitioners, and community volunteers to maintain awareness of the risk factors that affect child and family health [17]. Moreover, clear articulation of WHCP competency in regard to community, child, and family health is critical, as most WHCPs in China are not pediatric specialists or experienced in well-child health work [18]; in fact, it seems that many WHCPs have developed their competencies through continuous clinical practice and workplace training [19] and, therefore, they follow the Well-child Health Guidelines without possessing a clear understanding of the importance of well-child health care delivery [20]. Although the well-child care service has been provided in China for eight years, its quality and related factors, such as WHCPs’ perspective of well-child care and their role in providing the service, the type of service they provide, and the strategies and approaches used to promote the provision of care services, remain unknown. Considering this, we chose to conduct this qualitative study in order to obtain WHCPs’ views on well-child care and their role in providing such services, and to identify the factors that affect the quality of well-child care service provision and potential impediments to providing good-quality care.

## Materials and methods

### Study design

This study used a qualitative descriptive design. The final sample size reflects continuous sampling until the data were saturated (i.e., the point when most participants expressed similar views, or when no new ideas or information emerged during the interview process). Ultimately, our sample consisted of 22 WHCPs, who we interviewed concerning the well-child health care provided in community health centers in Hunan Province, China.

### Ethics statement

Ethical approval was obtained from the Medical Ethics Committee of The Third Xiangya Hospital, Central South University. Participants received an explanation of the study’s goal and were informed that participation was voluntary and that agreeing or declining to participate would not affect their works, family life, social relations, or salary. Participants were also assured that their individual responses would be confidential and that their anonymity would be preserved during data reporting and analysis. Consequently, all participants gave verbal informed consent to participate in this study (written consent was not obtained because we had mailed invitation letters concerning the study to the WHCPs; if the participant agreed to participate in the interview, he/she replied to the letter); furthermore, prior to the interview, the researchers reconfirmed the participants’ consent verbally. Participants’ statements of consent were recorded in the demographic questionnaire. This consent procedure was approved by the ethics committee.

### Participants

Participants were recruited from government community health centers between October 2014 and the end of January 2015. All targeted participants worked in a variety of community health care settings located in nine cities in Hunan Province. Hunan Province is divided into four main areas (Chang-Zhu-Tan Area, Southern Hunan Area, Western Hunan Area, and Dongting Lake Area) based on different economic levels and service levels. In order to ensure the representativeness of the sample, at least one city from each area was selected. Consequently, 50 participants working at 50 health care centers in the nine cities were invited to participate in the interview; of these, 30 agreed to participate in this study, and of these, five did not meet the inclusion criteria. Specifically, the inclusion criteria were as follows: location, ≥ 12 months of experience in well-child care, Chinese nationality, aged ≥218 years, ability to read and write Chinese fluently. Due to data saturation after interviewing the 25 participants, the final sample size was reduced to 22. The authors were unacquainted with the participants. Most WHCPs in China are female; accordingly, 72% of our participants were female (16 participants); all participants worked full-time.

### Data collection

Data were collected using semi-structured interviews, with participants completing a demographic questionnaire prior to completing the interview. Interviews were conducted face-to-face in an isolated room. Following a literature review, we developed five questions to serve as a guide for facilitating conversation with participants (See Table 1); this interview guide was tested on three WHCPs and revised accordingly before it was used in the interviews. The revised interview guide was critically examined by a multidisciplinary team of five experts (three well-child care experts, one public health specialist, and one pediatrician) who identified questions that were poorly worded, offensive, or indicative of the researcher’s bias or personal values. In order to collect the maximum amount of data, the questions were open-ended. Question-type sentences were used to encourage participants to provide additional useful information (e.g., *Would you give me some examples*? *Do you have any other suggestions concerning well-child care*? *Would you explain that further*?). The participants were permitted to freely express their views or suggestions on the main topic, and the researcher who conducted the interview then asked further questions based on the participants’ responses. Therefore, some variations in the participants’ views about the main topic emerged; if any information provided by the participants was completely unrelated to the topic of research, the researcher returned the conversation to the main topic by asking topic-specific questions once the participants had finished expressing their opinions.

**Table 1:** Interview question guide.

WHCPs’ perception of the well-child health care services they provide
1. Please describe the services you provide for children.
2. What other services do you provide for children and families through collaborating with others?
3. How do you consider your role and the overall well-child care service in your community health center?
4. What constraints do you encounter while providing services to children and families?
5. Recently, health care reforms in China have led to numerous changes. From your perspective, what should be done to improve well-child care service quality in community health care centers?

In order to protect the participants’ privacy, only two researchers and the participant were present during each interview. One researcher interviewed the participant, and the other researcher observed the participants’ expressions and noted key information. Interview duration was 25–30 minutes. All interviews were audio recorded.

Data collection continued until subsequent information was found not to contribute substantially to the understanding of the decision-making process. Once each interview ended, the recordings were immediately transcribed so that the researcher could summarize the participants’ perspectives of the well-child care service, and also so that they could identify interview questions for which data were still lacking. This latter information could then be collected during the interview with the next participant.

### Data analysis

The final sample size reflected our continuous sampling until saturation. The transcribed data were coded using Microsoft Office Word 2007, linking participants’ responses with the interview guide, and were analyzed by the research team using a descriptive content analysis method that comprised the following main phases: preparation, organizing, and reporting [21–23]. We began the preparation phase by selecting the unit of analysis; depending on the research question, the unit could be a word, letter, sentence, or a portion of text. The research team then worked to interpret the data and learn “what was going on” [24]. Next was the organization phase, which comprised performing open coding, abstraction, and creating categories. Open coding means that notes and headings were added to the text while reading it; the written material was thoroughly reread and as many headings as required were written along the margins in order to describe all aspects of the content. The headings were then collected in coding sheets, and categories were freely generated at this stage. After open coding, the lists of categories were then grouped under higher-order headings. The aim of grouping data was to reduce the number of categories by collecting similar or dissimilar observations into broader, higher-order categories. The data did not simply bring together observations that were similar or related; the data were classified as “belonging” to a particular group; this implies a comparison between certain data and other observations not belonging to the same category. The categories were formulated in accordance with the content of the interview guide. Next, in the abstraction phase, the research team collaboratively generated further categories to formulate a general description of the research topic. Finally, the research team organized and reported the analysis’ process and results.

## Results

### Participants’ demographic characteristics

Table 2 presents the participants’ characteristics. Most participants worked full-time, and all had > 5 years’ general work experience. The majority had an associate degree; in China, two levels of training are required to become a physician or a pediatrician: one is a bachelor’s degree and the other is an associate degree, which requires three years of college study after graduation from high school. The process for graduates from technical secondary schools is similar to that for high school graduates; however, the former must receive three years of education after graduating from junior high school (this rule has recently been abolished for prospective physicians, but it is still applicable for nurses). All physicians or pediatricians must also pass the clinical practitioners examination and obtain a certificate before they can begin working. Of our sample, three participants had a bachelor’s degree. For more individual information of the participants, please see the S1 File.

**Table 2:** Participants’ demographic characteristics (n = 22).

Characteristic	n (%), M (SD)
**Gender**	
Male	6 (22.3)
Female	16 (77.7)
**Age** (years)	38.9 (7.6) (27–56)
**Ethnicity**	
Han ethnicity	15 (68.2)
Hui ethnicity	5 (22.7)
Tu ethnicity	2 (9.1)
**Educational background**	
Bachelor’s degree	3 (13.6)
Associate degree	15 (68.2)
Technical secondary school	4 (18.2)
**Profession**	
General practitioner	2 (9.1)
Nurse	14 (63.6)
Pediatrician	6 (22.3)
**Work years**^a^	
< 6	0
6–10	14 (63.6)
> 10	8 (36.4)

^a^ “Work years” refers to the interviewee’s years of general work experience as a member of the medical staff of a community health center.

### Service content offered by participants

The majority of participants reported that they provided most of the care described in the 2011 Well-child Health Care Guidelines (i.e., immunization, routine health checks, family visits, high-risk-child management, hemoglobin screening, health education, and health follow-ups); these services were more frequently administered because they were free of charge. As a result of funding and resource limitations, most participants stated that they could not provide micro-element analysis, bone-density detection, or psychological counseling and intelligence assessment services; however, these services are more frequently required because some parents living in urban cities have only one child; one-child parents generally pay more attention to the growth and health of their child and, consequently, they may be more motivated to seek health care for their child. Two participants mentioned providing infant swimming and massage services, while one participant explained that their center practiced traditional Chinese outpatient medicine for children, permitting children to receive Chinese acupressure (we acknowledge that these modalities are not generally scientifically accepted outside of East Asia, and more research is required to prove their effectiveness and scientific nature). Several participants’ comments are presented in S2 File.

#### Health promotion as a key element of care provision

Participants highly evaluated health promotion in well-child health care; here, they generally emphasized health care consciousness, breastfeeding, and immunization. Most participants taught breastfeeding techniques to parents during newborn visits or routine physical exams, and all participants indicated that the most successful aspect of well-child health care delivery is immunization. The majority of the participants emphasized the importance of parents’ awareness of the benefits of preventive care; however, participants also reported that some parents remain unaware of the importance of some areas of well-child health care. Thus, overall, it seems that parents’ approach to health promotion is reactive. Participants did not discuss routine health checks or family visits, as these forms of care are typically emphasized in parental schooling, classes for mothers, TV and radio broadcasts, brochures, and promotion bars. Several participants’ comments are presented in S2 File.

### Working with other health professionals

The majority of the participants mentioned proactively collaborating with other health professionals (e.g., GPs working in the chronic disease management department, clinical laboratory physicians and nurses working in other departments); however, they seldom collaborated with other well-child health care providers, mainly as a result of differing responsibilities. In fact, two participants reported that, although they collaborated with two or three people to provide well-child health services, only one of the people actually performed the work. Some participants mentioned liaising with community and kindergarten care services, and others reported that their community’s neighborhood committee helped them to provide effective well-child care. This “neighborhood committee” refers to residents’ committees in communities; “neighborhood committees” can also play a promotional and supporting role in the delivery of well-child health care.

### Barriers to delivering well-child health care

#### Barriers affecting WHCPs

The participants mentioned barriers affecting service provision at both individual and system-wide levels. All participants identified inadequate training and time as the main barriers to delivering well-child services, and they also mentioned that their salary was too low to meet their living costs. Most WHCPs reported that their salary was 2000–3000 RMB, which is much lower than the average annual wage of staff and workers in similar sectors (2014) in Hunan Province (over 4000 RMB), and the low salary also resulted high drop-out rate of WHCP.

#### Barriers affecting the health care system

Participants reported the following factors as core systemic barriers to providing well-child health care services: inadequate WHCP-specific knowledge and funding, inspection equipment, and collaboration with supervising departments; insufficient attention from the community manager; and inadequate information-sharing support.

The most participants thought that the principal limitation to well-child care delivery was determined to be inadequate professional knowledge of well-child care. Further, due to the high drop-out rate among WHCPs, we found that some community centers are unable to find pediatric specialists to provide well-child care services; consequently, the leaders of community health care centers are forced arrange for medical staff from other work departments to perform these tasks; for example, members of the chronic disease management department or the women’s care management department. For the first few months of their careers, these medical staff are un-experienced and must learn well-child care knowledge by themselves. Concurrently, they are required to continue their training in well-child care by attending a superior mental and child health care department, and also to complete certification exams in order to obtain a WHCP certification.

The participants also reported experiencing difficulty securing adequate time to assess children and to communicate with parents about health promotion, and also states that they spent most of their time updating health records. Some participants also mentioned that the management of “floating children” is another barrier to their work. Floating children refers to children who formerly lived with their parents in another city and do not have a registered permanent residence certification for the city they presently live in, or children who have frequently changed their place of residence.

### Facilitating factors

The majority of the participants believed that more targeted training concerning well-child care issues and related skilled techniques would significantly facilitate the delivery of well-child care services. Further, they mentioned their lack of well-child-health-care-specialized knowledge and experiencing distrust from families as impediments to service delivery, suggesting that specialized training interventions would improve the situation by increasing WHCPs’ knowledge and developing their skills, thereby allowing them to develop more patience, responsibility, and compassion, which are necessary to successfully deliver well-child care services.

Some participants also believed that large-scale awareness campaigns could improve the delivery of well-child care services. They suggested that government and media publicity would increase families’ care awareness and their approval of well-child health care.

Moreover, participants recommended that the government set aside special funding for well-child care services, provide additional professional examination equipment for children (e.g., trace element detectors, jaundice monitors, and special vision detectors), and emphasize service quality over service quantity.

S2 File includes five topics identified from the participants’ answers: the service content, the views of WHCPs toward well-child care services, collaboration with other staff, barriers to delivering well-child health care, and facilitating factors of well-child care delivery. The specific questions and several participants’ comments are also presented in S2 File.

## Discussion

The majority of participants possessed a medical degree, but did not specialize in pediatrics; which indicates that, in order to improve well-child health care delivery in Hunan Province, reforms should focus on improving education and training for WHCPs’, as has been suggested in other regions in China [25–27].

This study’s participants reported that the well-child health care services they provided were below the standard recommended in the Well-child Health Care Guidelines. Further, it was reported that many parents prefer to visit main hospitals rather than community centers, even though community centers’ services are free. Parents’ satisfaction with their young child’s health care has been found to predict subsequent immunization, infant health status, and patterns of maternal health care usage, independent of socio-demographic characteristics [28]; thus, community centers should offer timely and age-appropriate well-child care, as this will encourage parents to bring their children to such centers more frequently. In the US, comprehensive services are provided to children and young adults aged < 19 years, whereas in China such services are only provided to children aged ≤6 years [12, 16]; consequently, it can be recommended that comprehensive health care services for children aged between six and 18 years be implemented in China’s this would greatly improve the health status of this demographic.

Building on the Well-child Health Care Guidelines, reforms should target current shortfalls in well-child care. None of this study’s participants reported performing mental health screenings. This is significant because studies have found that the prevalence of psychological and physiological implications in children is increasing [29–31]. Most relevant guidelines recommend that health care providers use standardized screening tools to assess suspected cases of poor mental health among children [32–34] (examples of such tools are the Ages and Stages Questionnaires—Second Edition (ASQ) [35] and the Ages and Stages Questionnaires—Social Emotional—Second Edition (ASQ-SE)) [36]. Mental-health screenings include examining self-regulation, compliance, communication, adaptive behavior, autonomy, affect, and interaction with others [37]. Considering this, it can be suggested that future reforms of well-child health care focus on providing early detection of delayed development, illnesses, and health risk factors, particularly emphasizing mental health.

This study’s participants described promoting the dissemination of health-promotion information as particularly important, but also reported that their care was typically limited to immunization, health education, and promoting breastfeeding, rarely addressing the parent-child relationship. In China, the importance of the parent-child relationship is somewhat poorly understood; grandparents traditionally care for young children, and most young parents quickly return to work following their child’s birth; this partly accounts for the increasing number of children left at home alone. Also notable is that grandparents are less likely than parents to closely monitor their relationship with children and to cooperate with WHCPs, and that the number of left-behind children is increasing, which is resulting in further pressure being placed on well-child care delivery [38,39]. Ironically, international research indicates that routine visits to WHCPs promotes monitoring of family dynamics [40,41].

Health professionals should work together in order to promote the delivery of well-child care [42]. Studies have suggested that well-child care teams can more effectively provide care services to children and families [43,44]; such teams typically include a general practitioner, nurse, and WHCP.

At the individual level, lack of time is among the most important barriers to the optimal provision of WHCP care. Chinese studies have indicated that most pediatricians and other health providers do not have sufficient time to deliver appropriate well-child care [27,45]. Thus, a practice nurse may be greatly beneficial in such circumstances, as they can provide comprehensive primary physical examinations and record changes in health.

At the system-wide level, inadequate funding is among the most important barriers to adequate well-child care provision. At present, the Chinese government allocates each resident 35 Yuan (RMB) annually to pay for public health care services. This funding is intended for all services, such as chronic disease management, maternal health care, well-child care, and mental disturbance management [46]. However, no funds are specifically allocated for well-child care services. Of course, we should recognize that national resources are always limited, and greater funding for one program means reduced funding for other programs; in other words, elected officials must balance the demands of many different agencies and programs. Nevertheless, the US has established a special child health insurance program; children covered under this program may access free health care, with the fees being paid by the government and insurance companies [47]. This program could represent an example for China to create a system to provide children with access to high-quality care services.

### Limitations

All of this study’s participants were recruited from urban primary community centers; we did not examine WHCPs from primary health care clinics located in rural areas. Consequently, this limits the present results’ generalizability to other care settings. Second, despite the data being saturated after interviewing 22 participants from four different economic levels and service level cities in Hunan Province, resulting in the discovery of new ideas and information, the sample size was small.

Next, we featured open-ended questions in the interviews in order to obtain more information on participants’ opinions on well-child care services; this may have led to unwanted information being collected and measurement bias. However, if the information provided by the participants was completely unrelated to the topic of research, once the participant had expressed their opinions, the researcher redirected the conversation to the topic by asking topic-specific questions.

Finally, this study did not evaluate parents’ satisfaction with well-child care outcomes.

Despite the above limitations, the present findings may inform and guide reforms aiming to improve the content and delivery of well-child care.

Future research should examine WHCPs’ role in health care clinics in rural areas of Hunan Province. Further, including parents’ perspective on well-child health services’ delivery may enhance the understanding of WHCPs’ role in delivering health care services, consequently improving those services’ delivery.

This study only analyzed the well-child service in urban cities; future, larger studies could focus on both urban and rural centers, comparing the well-child care services provided in both areas.

## Conclusion

This study extends the understanding of WHCPs’ role in providing well-child health services and WHCPs’ perspective on the current state of the well-child care system. We consequently identified the limitations of the well-child care system in urban China. Specifically, these limitations are as follows: inadequate WHCP-specific funding, inspection equipment, information-sharing support, and collaboration with superior support, as well as insufficient attention from community managers. These limitations can be addressed as follows: WHCPs, as key service providers, should collaborate more with other health professionals and related staff in the community; community health center managers should aim to overcome systemic barriers to care delivery; and WHCPs should aim to extend their knowledge and expertise in order to facilitate their care delivery.

## Supporting information

S1 File The individual data of participants. (DOCX)

S2 File The Questions and participants’ comments. (DOCX)
