# Supplementary material for: Well-child care delivery in the community in China: Related factors and quality analysis of services
Source: PLoS One. 2018 Jan 23;13(1):e0190396. doi: 10.1371/journal.pone.0190396 (PMC5779649; doi:10.1371/journal.pone.0190396)
Supplement: S1 File — (DOCX) [file pone.0190396.s001.docx]

S1 File The individual data of participants

| Number | City | Gender | Age  (years) | Nationality | Educational level | Profession | Working years in community center |
| --- | --- | --- | --- | --- | --- | --- | --- |
| 1 | Changsha | Female | 48 | Han | Associate degree | Nursing | 28 |
| 2 | Shao  yang | Female | 36 | Han | Technical secondary school | Nursing | 10 |
| 3 | Xiangxi | Male | 56 | Tujia | Associate degree | General Practitioner | 34 |
| 4 | Changsha | Female | 33 | Han | Associate degree | Nursing | 8 |
| 5 | Changsha | Male | 34 | Han | Associate degree | Pediatrician | 9 |
| 6 | Xiangtan | Female | 35 | Han | Associate degree | Nursing | 10 |
| 7 | Xiangtan | Female | 32 | Hui | Associate degree | Pediatrician | 9 |
| 8 | Yue  yang | Female | 36 | Han | Bachelor’s degree | Pediatrician | 10 |
| 9 | Yue  yang | Female | 43 | Han | Associate degree | Nursing | 19 |
| 10 | Lou di | Female | 37 | Hui | Bachelor’s degree | Nursing | 10 |
| 11 | Xiang  tan | Female | 27 | Han | Technical secondary school | Nursing | 6 |
| 12 | Zhangjiajie | Female | 49 | Hui | Associate degree | Nursing | 26 |
| 13 | Yong  zhou | Male | 44 | Han | Associate degree | Pediatrician | 20 |
| 14 | Lou  di | Male | 45 | Han | Bachelor degree | Pediatrician | 21 |
| 15 | Yue  yang | Male | 36 | Han | Associate degree | General Practitioner | 10 |
| 16 | Chang  de | Male | 45 | Hui | Associate degree | Pediatrician | 25 |
| 17 | Yue  yang | Female | 34 | Han | Technical secondary school | Nursing | 10 |
| 18 | Xiang  tan | Female | 30 | Han | Associate degree | Nursing | 8 |
| 19 | Lou  di | Female | 48 | Hui | Technical secondary school | Nursing | 25 |
| 20 | Yong  zhou | Female | 30 | Tujia | Associate degree | Nursing | 8 |
| 21 | Chang  sha | Female | 39 | Han | Associate degree | Nursing | 10 |
| 22 | Yong zhou | Female | 39 | Han | Associate degree | Nursing | 9 |
