# Supplementary material for: Well-child care delivery in the community in China: Related factors and quality analysis of services
Source: PLoS One. 2018 Jan 23;13(1):e0190396. doi: 10.1371/journal.pone.0190396 (PMC5779649; doi:10.1371/journal.pone.0190396)
Supplement: S2 File — (DOCX) [file pone.0190396.s002.docx]

S2 File . The Questions and participants’ comments

| Topic | Questions | Participants’ answers(selected partly) |
| --- | --- | --- |
| **Services content** | 1. What services do you provide for Well-child Care?  2. Do you provide any other paid services in addition to the regular basic services provided free of charge?  3. Are there any services you don’t provide but the parents may wish to do it? And why did you not provide these services? | *“Every time they came to our center, I did a physical examination of the child including height, weight, head circumference, chest circumference, vision, and hearing. In other words, I did the work according to the Well-child Health Care Guidelines.”(N1)*  *“We have provided traditional Chinese medicine in addition to the basic physical examination. Services include acupressure, Chinese medicine (Jianpi pills), and muscle kneading. However, the effects were not optimal because the parents couldn’t adhere to it or couldn’t find the acupuncture points accurately.”(N3)*  *“We do not provide microelement analysis or mental health care management because we do not have the equipment. If the parents would like to do it, we suggest that they visit the municipal maternal and child care service hospital.”(N7)* |
| **The views of WHCPs toward Well-child Care** | 1. What do you think of the Well-child Care services in your community?  2. In your opinion, what services are most successfully provided in your community center?  3. According to you, what is the most important thing to promote the Well-child Care service delivery? | *“Every time they brought the child here, I asked the parents about their child’s feeding situation. I discussed the introduction of solid food with parents if their child was aged over six months. Sometimes, if they came across any questions regarding child feeding, they would telephone me and I would answer their queries.” (N12)*  *“During their first visit, I would tell the mother how to breastfeed the baby properly. The mother listens to me carefully” (N20)*  *“As soon as it was time for their child’s immunization, the parents would come here on time. Even if they couldn’t come here on the exactly, they would get their child immunized another day.”(N16)*  *“Generally speaking, the parents came for immunization on time. If they forgot, we would call them.” (N21).*  *“If parents pay more attention to preventive care, we can deliver the service more effectively.” (N15)*  *“In the recent years, parents’ perception of health care has been greatly improved due to increased publicity of well-child health care.”* (N8) One participant reported that *“The first thing I think of is improving the parents’ perception of health care. According to them, Well-child Care is just vaccination. They are unwilling to visit well-child health care centers to get physical exams and other services. This is especially true for caregivers who are grandparents. They do not receive enough education and cannot comprehend the importance of Well-child Care.” (N7)*  *“We made many brochures for the parents, and had a bulletin board, but parents just read the content, rather than routinely practicing the methods taught in the brochures.” (N3)*  *“We organized a workshop for mothers and invited a health professor to give a lecture, but the majority of parents were unwilling to attend.” (N13)* |
| **The collaboration with other staffs** | 1. Will you collaborate with others during Well-child Care delivery? 2. What services do you provide together? 3. How do you collaborate with others? | “*We usually work within our own departments. I would refer to the physician or the GP only if there was a problem that I could not solve.” (N18)*  *“Every year we go to the child care center to provide health care services for children aged above 3 years. There were so many children and too much work; during such times, we worked with other health professionals, such as physicians, practice nurses, and public health care workers.” (N9)*  *“There is a nurse who helps me with the physical examination, but she is a new hand. She does not know how to conduct the examination accurately. She does not even know the normal range of growth and development of children, so I have to do that by myself.” (N7)*  “*We only have one WHCP. I do everything myself, including physical examination, electronic health records, and health education. There are several things to do and sometimes I cannot meet the standards. However, since the superior maternal and child health center evaluate my performance at regular intervals, I have to fudge the data.” (N14)* |
| **Barriers to delivering well-child health care** | 1. What constraints do you encounter while providing services to children and families? 2. Can you express the constraints more clearly? 3. Do you think the constraints are caused by yourself or other aspects? | *“There are too many things. I do not have time and readiness to always treat efficiently. On physical examination days, I need to see more than 20 children in one morning. It is impossible to conduct examination in detail.” (N19)*  *“Sometimes I don’t feel very confident in resolving a mother’s concerns about her child’s development because I majored in nursing. I did not receive specialized training before I started working in well-child health care, and there are some content that I am not very clear on. I have obtained competence and experience through daily work and routine training in later work. (N20)”*  *“As far as I am concerned, my salary is much lower than that of a nurse. The turnover rate of WHCPs was very high and they would not work there for a long time.” (N1)*  *“...not only our center, it is almost all centers. The hardware facilities can hardly meet the demands of the care service... Second, the community center manager does not attach much importance to Well-child Care. They prefer to use services that bring economic benefit.” (N15)*  *“In my opinion, we should work together with advanced settings to strengthen our connection. Usually, I suggest that children visit the superior hospital, but that hospital does not provide feedback about the child’s health to us; therefore, we cannot get continuous information about the child.” (N14)*  *“The information platform cannot be shared with other community centers. If the child receives a physical examination in another center, we cannot get the information to complete the electronic health records. This is another reason why we have to fudge the data.” (N22)*  *“I think the first thing is to set specific funding for Well-child Care services in community centers. The wage is too low to support our lives, therefore, many WHCPs are unwilling to do the work in community centers, and WHCPs’ mobility is very high.” (N21)*  *“There are too many floating children in our community. Sometimes their parents bring them out to work or leave them at their grandparents’ home. We cannot prepare health records and provide well-being service to the children on time. That can cause trouble for our work.” (N12)* |
| **Facilitating factors of Well-child Care delivery** | 1. Recently, health care reforms in China have led to numerous changes. From your perspective, what should be done to improve Well-child Care service quality in community health care centers? 2. What should you and your community center do to promote the Well-child Care delivery? 3. What should the government and social public do to promote the Well-child Care delivery? | *“I think specialized training in Well-child Care services, such as feeding, child psychology, prevention of accidental injury... and a step-by-step standard physical examination process could be easily incorporated into my daily practice. The training is also an easily accessible informational resource I can trust.”(N10)*  *“In my opinion, it’s important to strengthen the training of well-child health care providers, especially in practical skills. I think if we can have on-the-job training at a superior hospital, we can learn more practical skills.” (N7)*  *“I think it’s necessary for a well-child health care provider to have compassion and patience in their work, so that families will be willing to trust you and cooperate with you.”(N11)*  *“TV spots and public service announcements may be an effective way to increase the awareness of Well-child Care services.” (N8)*  *“The government also needs to work with us to publicize Well-child Care services. It is difficult to change the attitudes of families without [the governments’] assistance. They are more likely to believe the government’s policy.”(N14)*  “*I think there are too many areas that need to be improved in Well-child Care services. The government should invest more funds and provide more equipment for community Well-child Care.” (N22)*  *“The standard rates of well-child health care management are too high for us. Sometimes there are not many children suffering from anemia in our community; however, the guidelines stipulate that the rate of anemia management must be reached. We cannot reach the standard at all; therefore, we fudge the data sometimes. I hope the superior settings take service quality into greater consideration, and not the quantity.” (N16)* |
